# Over-specification of small, borderline cardinalities and color in referential communication: the role of visual context, modifier position, and consistency

**DOI:** 10.3389/fpsyg.2024.1417047

**Published:** 2024-07-29

**Authors:** Natalia A. Zevakhina, Kseniya N. Dongarova, Daria Shubina, Daria P. Popova

**Affiliations:** HSE University, Moscow, Russia

**Keywords:** over-specification, referential communication, cardinalities, color, modifier position, consistency

## Abstract

This paper reports on two flash-mode experiments that test redundant descriptions of small (2–4) cardinalities, borderline (5–8) cardinalities, and color in referential communication. It provides further support for the idea that small cardinalities are more salient (due to subitizing), less sensitive to visual context, and therefore give rise to higher over-specification rates than color. Because of greater salience, Russian speakers more often use prenominal positions for numerals than for color adjectives. The paper also investigates borderline cardinalities and argues for the order factor that affects their salience, since ordered items can be perceived in small subitized parts. The ordered mode of presentation of the borderline cardinalities leads to higher over-specification rates and to higher percentages of prenominal positions than the unordered one. The paper provides further evidence for the consistency of small, borderline cardinalities, and color in people’s choices to minimally specify or over-specify given objects in referential communication.

## 1 Introduction

### 1.1 Over-specification of properties in referential communication

It has been acknowledged that interlocutors often communicate redundant information when referring to objects shared in a visual environment. When the speaker sees a set of different objects and is aware that this set is identical for the listener, the speaker might refer to an object merely by naming it. To illustrate, in a situation where interlocutors see a cup, a plate, and a spoon, it is sufficient for the speaker to communicate (1).

1. *Give me the cup, please*.

Another way of referring to a cup in the same context is to indicate its property, e.g., color. Suppose that the objects differ in color: a red cup, a green plate, and a yellow spoon. The speaker might produce an utterance (2) in which she provides a more detailed description of an object.

2. *Give me the red cup, please*.

Strictly speaking, to articulate a color adjective is to articulate a redundant piece of information, which is not needed for the purpose of communication since the correct object can be identified by interlocutors in a simpler way (1). If it is, why does the speaker report an unnecessary property in her message?

At first sight, a redundant linguistic expression violates the Gricean maxim of quantity that says: ‘Do not provide more information than is required’ (Grice, 1975). In other words, the speaker should not provide over-specified descriptions since they are over-informative for the listener. Does it make communication easier for the speaker or more efficient for the listener? One possible answer to this question takes a speaker-oriented perspective (Pechmann, 1989; Brown-Schmidt and Konopka, 2008; Wu and Gibson, 2021). According to this account, the speaker articulates the information that catches her eye while examining a visual context. In some other approaches neutral to the account under consideration (Belke and Meyer, 2002; Koolen et al., 2011; Tarenskeen et al., 2015; among others), color has been argued to be the best choice to communicate due to its easy and early discriminability and high salience. Color is usually not compared to some notional standards or competitors given in a visual context. It has an absolute meaning that is not context dependent (Pechmann, 1989; Belke and Meyer, 2002), but see a recent study by Hansen and Chemla (2017).

Another possible answer to the question is that a redundant expression makes communication more efficient for the listener and, consequently, is listener-oriented (Mangold and Pobel, 1988; Clark and Schaefer, 1989; Rubio-Fernandez, 2016; Long et al., 2021; Rubio-Fernandez et al., 2021; Rubio-Fernandez, 2021; Jara-Ettinger and Rubio-Fernandez, 2022). According to this approach, redundancy in referential communication conforms to the Gricean principle of cooperation (Grice, 1975), since efficient messages communicated by the speaker enable the listener to identify an object under discussion easily and rapidly. As mentioned above, color is perceived as a salient and absolute property of an object (Belke and Meyer, 2002; Koolen et al., 2011; Tarenskeen et al., 2015). The speaker knows that color is absolute and salient and expects the listener to be also aware of this. This line of reasoning induces the speaker to articulate a color linguistic expression, although this is redundant.

Besides color, other properties have also been investigated, namely size, shape, material, pattern, location, orientation (Belke and Meyer, 2002; Engelhardt et al., 2006; Arts et al., 2011; Brown-Schmidt and Konopka, 2011; Koolen et al., 2011; Gatt et al., 2013; Van Gompel et al., 2014; Tarenskeen et al., 2015; Rubio-Fernandez, 2016, 2019, 2021; Long et al., 2021; Rubio-Fernandez et al., 2021; among others). The studies reported that these properties are less salient than color. Some of them are relative and context dependent. Size is a good example of lesser salience and context dependence. Its discriminability depends substantially on the environment. Consider a visual context with a big cup, a big plate, and a big spoon. If the interlocutors share this identical set of objects and are mutually aware of this, the speaker is more likely to choose a minimalistic expression (1) than to produce a redundant expression (3).

(3) *Give me the big cup, please*.

Consider now a context with objects that differ in size: a big cup, a small plate, and a medium-sized spoon. If interlocutors share this identical set of objects and are mutually aware of this, the speaker might consider that the shared visual context is contrastive and is more salient for her and her listener. This increases the probability that the speaker specifies a size linguistic expression as in (3), even though it is redundant.

The general aim of the present study is to investigate whether cardinality, a less known property of a set of objects, is over-specified in referential production and how it interacts with color, the most salient and absolute property of objects, according to the literature (as said before). The paper is structured as follows. The rest of Section 1 discusses recently discovered relevant facts about cardinalities and color. Section 2 formulates the research questions and hypotheses of the present study tested in two experiments and reported in Sections 3 and 4, respectively. Section 5 discusses the main contributions of the paper, and Section 6 concludes the paper.

### 1.2 Cardinalities, subitizing, and over-specification

A cardinality is a numerosity of some set of objects, that is, a quantity of items in a set. To illustrate, five elephants represent a set of elephants with a numerosity of five. One of the facts about cardinalities relevant to the present study is subitizing. Subitizing is a human capacity to instantaneously grasp some (but not all) cardinalities and is opposed to estimation (Trick, 1992; Bourdon, 1908; Stevens, 1938; Taves, 1941; Kaufman et al., 1949; Thomas et al., 1999; Revkin et al., 2008). Subitizing has been argued for small cardinalities up to four, and it lacks in large cardinalities greater than 8 that involve estimation. This suggests that humans possess two separate cognitive mechanisms (Revkin et al., 2008). One is for subitizing that is rapid, accurate, and effortless. It usually takes 40–100 ms per item. Another one is for estimation, which is effortful, error-prone, and slow. It usually takes 250–350 ms per item (Trick, 1992). The borderline zone of cardinalities 5 to 8 is highly dependent on individual capacities and varies from person to person; it also depends on the experimental setting and some other factors (Jensen et al., 1950; Chi and Klahr, 1975; Atkinson et al., 1976; Akin and Chase, 1978; Oyama et al., 1981; Mandler and Shebo, 1982). Both mechanisms, subitizing and estimation, are distinct from counting that is determined by Stevens (1938, p. 95) in the following way: ‘In order to specify the numerosity of any group we have merely to pair successively each object in the group with a numeral from the numeral-series, beginning of course with the first numeral in the series.’ However, Piazza et al. (2002) provided PET experimental evidence against the idea that subitizing and counting are implemented as two separate neural processes. Moreover, that paper revealed an effect of order/arrangement of subitized/small (1–4) cardinalities vs. higher/borderline (6–9) cardinalities in terms of reaction times. The canonically and randomly arranged subitized cardinalities were processed similarly, whereas the randomly arranged borderline cardinalities were processed longer than the canonically arranged ones. Margolis (2020) argued for an innate small number domain-specific system that does not involve counting, takes into consideration not only visual but also auditory input, and accounts for the capacity limit on infants’ working memory. Martí et al. (2016) showed that the development of small cardinalities depends on a cognitive task children face with and on a socioeconomic environment.

Although small (2–4) cardinalities are considered redundant pieces of information, Zevakhina et al. (2021) argued that they are over-specified in referential communication due to subitizing that makes them salient. Regarding salience, small (2–4) cardinalities resemble color (Brown-Schmidt and Konopka, 2011; Zevakhina et al., 2021).

Cardinalities are linguistically expressed by numerals. Languages tend to distinguish between small numerals (up to 4) and numerals larger than 4 in various ways (see Hurford, 2001 for details). The first evidence comes from the grammatical number system that goes beyond the well-acknowledged single-plural distinction in nouns. For example, Arabic has a dual grammatical category for nouns. More generally, as Overmann (2015, p. 641) showed, ‘concepts of and words for subitizable quantities may emerge first because subitizable quantities are more perceptually salient.’ Despite the general pattern that a lexical numeral system emerges prior to a grammatical number system, the emergence of number for subitizable and higher quantities is quite similar in both systems: lexical numerals and grammatical number categories for subitizable quantities are observed earlier than those for higher quantities (Overmann, 2015). Corbett (2000) argued that dual and trial grammatical forms (forms of subitizable quantities) originate from numerals ‘two’ and ‘three’ (see also Greenberg, 1972).

The second evidence comes from the fact that nouns denoting small and large cardinalities can have different number grammatical categories, see Section 2.2 for a distinction between small and large numerals in terms of number (and case) grammatical system.

It has been argued that numerals have two meanings: lower-bound meanings ‘at least n and possibly more’ and upper-bound meanings ‘exactly n’ (*cf.* Papafragou and Musolino, 2003; Musolino, 2004; Breheny, 2008; among others). Lower-bound meanings are primary, and upper-bound meanings are derived from them via the scalar implicature mechanism. It is reasonable to say that subitizing involves a precise perception of small (2–4) cardinalities and that the numerals to which they correspond have upper-bound meanings. Suppose that there are three cups on the table and suppose that the speaker has an intention to ask the listener to pass her all three cups. The speaker is more likely to communicate (4) by conveying the upper-bound meaning ‘three cups exactly’ than (5) by conveying the lower-bound meaning ‘at least two cups and possibly three’. Upper-bound meanings are not relative, and, therefore, they are absolute.

4. Give me three cups, please.
5. Give me two cups, please.

### 1.3 Over-specification of color and small cardinalities

It is well-known that color demonstrates higher discriminability and higher percentages of over-specification when the environment includes items of different colors. Polychrome contexts, unlike monochrome ones, increase redundant color expressions (see Belke and Meyer, 2002; Koolen et al., 2013; Rubio-Fernandez, 2016; Rubio-Fernandez et al., 2021; Long et al., 2021; among others). Section 1.1 illustrated a polychrome context with a red cup, a green plate, a yellow spoon, and the utterance (1). Analogously, an example of a monochrome context would be a red cup, a red plate, and a red spoon. Interestingly, Zevakhina et al. (2021) argued that small (2–4) cardinalities are over-specified significantly more often in multi-cardinality contexts than color in polychrome contexts. By multi-cardinality contexts we mean visual contexts in which, for example, there are two cups, three plates, and four spoons. They contrast with one-cardinality contexts that can be illustrated by two cups, two plates, and two spoons. Interestingly, when the environment includes items of an identical cardinality and of an identical color, either both properties (color and cardinality) were articulated, or only cardinality was, as Zevakhina et al. (2021) showed. To illustrate, if there are two red cups, two red plates, two red spoons, the speaker would rather produce (6) than (7) or (8).

6. Give me two cups, please.
7. Give me two red cups, please.
8. Give me red cups, please.

We see from the findings given above that cardinality is over-specified significantly more often than color in various visual contexts, presumably because cardinality is more salient than color.

### 1.4 Prenominal and postnominal positions of color modifiers

Both speaker-and-listener-oriented approaches (see references in Section 1.1) emphasize that utterances are produced by the speaker and processed by the listener incrementally. It means that production and processing of words occur in a linear and incremental way as they are encountered in linear time (see Kachakeche et al., 2021, p. 3006). The order of words in the language reflects this. From the speaker’s viewpoint, the information that is produced earlier is more salient for the speaker and, as the speaker believes, is more efficient to guide the listener’s visual search for an intended referent. From the listener’s viewpoint, the information that takes a linearly preceding position in the speaker’s utterance is considered by the listener as more salient and more efficient and assists the listener to find an intended referent. In this regard, as the literature has demonstrated (Rubio-Fernandez, 2016; Rubio-Fernandez, 2019; Rubio-Fernandez et al., 2021), prenominal color adjectives are more efficient than postnominal ones. Consequently, English prenominal color adjectives are over-described to a greater extent than Spanish postnominal ones (*ibid.*). Note that Spanish allows for both postnominal and prenominal adjectives, but the Spanish adjectives considered in the cited papers are postnominal, and Spanish adjectives are prototypically (i.e., in unmarked position, with a higher occurrence) postnominal. According to Waldon and Degen (2021) and Yu et al. (2023), utterances with prenominal adjectives are assigned higher probabilities than utterances with postnominal adjectives. A large-scale corpus-based study reported by Kachakeche et al. (2021) involved 74 languages and showed that languages with prenominal adjectives favor adjectival modification to a greater extent than languages with postnominal adjectives. This is an interesting result, as cross-linguistically, postnominal adjectives exhibit a more frequent phenomenon than prenominal adjectives (WALS; Dryer, 2013a).

Languages with prenominal adjectives reveal postnominal occurrences of adjectives in disfluent speech. As illustrated by Brown-Schmidt and Konopka (2008), English size adjectives disfluently occurred in the postnominal position (*the butterfly…uh small one*), much like Spanish size adjectives (*la mariposa pequeña*, literally ‘butterfly small’).

### 1.5 Consistency in referential communication

Several studies investigated the question of whether participants demonstrate consistent behavior during an entire experiment. Speakers can minimally refer to the items given in a visual context by naming them or providing additional characteristics of them, e.g., their color or cardinality. According to Brennan and Clark (1996), interlocutors make a temporary agreement on how they conceptualize objects in a visual context by using identical lexical items. Pickering and Garrod (2004) argued that interlocutors have interactive automatic alignment of representations at various language levels during a dialogue. Therefore, they use lexical repetitions in their speech. Goudbeek and Krahmer (2012) established connections between alignment and over-specification of properties in referential communication. Interlocutors usually name properties previously articulated in a dialogue. A more recent paper by Tarenskeen et al. (2015) demonstrated that speakers are usually consistent in describing given objects. They do so either in a minimalistic way, i.e., naming objects, or in an over-specification way, i.e., additionally naming properties of objects.

## 2 Present study

### 2.1 Research questions related to visual context and consistency

With all the above points in mind, the present study aims to answer the following questions.

Firstly, recall that small (2–4) cardinalities and color have much in common in terms of salience and absoluteness, but small (2–4) cardinalities were reported to be more often over-specified than color due to subitizing (see Sections 1.2 and 1.3). It is worth investigating the interaction of small (2–4) cardinalities and color in various visual contexts and the question of how this interaction affects production of corresponding linguistic expressions, that is, numerals and color items, in referential communication. Therefore, the present study poses the question of whether this tendency is preserved in the following two situations: (i) all objects are identical in terms of color and cardinality (that is, monochrome and one-cardinality contexts) and are presented in flash mode; (ii) all objects are different in terms of color and cardinality (that is, polychrome and multi-cardinality contexts) and are presented in flash mode. The flash mode presentation was aimed at imitating subitizing effects. Monochrome and one-cardinality contexts are visually neutral in both color and cardinality, while polychrome and multi-cardinality contexts are visually contrastive in both color and cardinality. The idea was to examine which property would be over-specified in neutral and contrastive contexts, that is, which property is more salient.

Secondly, borderline (5–8) cardinalities represent another area of interest. It has not been investigated yet whether they are redundantly included in descriptions of objects in referential communication (*cf.* Section 1.2). In this respect, it is interesting to study the following question: Does ordered vs. unordered presentation of 5–8 objects affect cardinality over-specification? The idea is that an ordered design is likely to facilitate perception of cardinality, since the objects are visually presented in small groups (2–4 items per group) that can be subitized. An unordered design seems to impede instantaneous grasping of a cardinality, and therefore perception of a cardinality might be error-prone. Therefore, the question is whether an ordered design gives rise to higher over-specification rates than an unordered one.

Thirdly, it is interesting to study whether consistency is observed in labeling objects (and their color and cardinality properties) throughout referential communication. The idea is that if the speaker either minimally or over-informatively refers to a given object, she does so during a whole experiment. Previous literature studied this with respect to colored objects (see Section 1.5). Now the question is about small sets of colored objects.

### 2.2 Research questions related to the position of adjectival and numeral modifiers

Based on previous research (see Section 1.4), one more question that deals with the position of adjectival and numeral modifiers has not yet been studied. The following question is worth investigating in a language with basic prenominal modification: Do adjectival or numeral modifiers reveal disfluency and are postnominally used? If so, does this exhibit a regular phenomenon? Are there differences between numerals and adjectives in this respect?

For current purposes, we took Russian, which was the only language available at the time of investigation. It is a language with the following basic prenominal word orders: ‘numeral + noun phrase’ in a numeral phrase and ‘adjective + noun phrase’ in a noun phrase with adjectival modification (WALS; Greenberg (ed.), 1963; Bivon, 1971; Dryer, 2013a,b). By a basic word order we mean a word order that is unmarked in terms of information structure and has higher frequencies.

Importantly, if a numeral takes the Nominative or Accusative case, it is in the Specifier position of a quantifier phrase, and the null head Q assigns the Genitive case to a noun phrase as its complement (Bailyn, 2004). A noun phrase in the complement of Q is assigned the singular form if the Specifier position of Q has a numeral of the meaning ‘two,’ ‘three,’ or ‘four,’ *cf.* (9). A noun phrase in the complement of Q is assigned the plural form if the Specifier position of Q has a numeral of the meaning ‘five’ or more than five, *cf.* (10). If a numeral takes other case forms, this case is assigned to a numeral and a noun phrase by a verb (*ibid.*). Since referential communication involves naming, the Nominative case for a numeral and the Genitive case for a noun phrase are expected to be used. Adjectives are adjuncts to noun phrases. They agree in case with a numeral. An adjective always has a plural form regardless of the number form of a noun phrase, *cf.* (9)–(11).

9. *Tri krasnyx šarfa*.

three.NOM red.GEN.PL scarf.GEN.SG

‘Three red scarves.’

10. *Pjat’ krasnyx šarfov*.

five.NOM red.GEN.PL scarf.GEN.PL

‘Five red scarves.’

11. *Dumaju o pjati/trjox krasnyx šarfax*.

think.1SG about five.LOC/three.LOC red.LOC.PL scarf.LOC.PL

‘I am thinking of three red scarves.’

### 2.3 Hypotheses of the present study

Based on the research questions formulated in Sections 2.1 and 2.2, the present study aims to verify the following hypotheses in two experiments.

According to Hypothesis 1, the over-specification of small (2–4) cardinalities is expected to be higher than the over-specification of color. This is motivated by previous research (Zevakhina et al., 2021), according to which small (2–4) cardinalities are more salient than color due to subitizing. We verify this hypothesis in the first experiment of the present study and view this, on the one hand, as a replication and, on the other hand, as an extension of the established effect but in a modified environment: in a flash-mode design with same vs. different color and cardinality conditions.

Hypothesis 2 says that visual context influences over-specification rates and is divided into Hypotheses 2A, 2B, and 2C. Firstly, relying upon the literature (Belke and Meyer, 2002; Koolen et al., 2013; Rubio-Fernandez, 2016; Long et al., 2021; Rubio-Fernandez et al., 2021), color over-specification is predicted to be higher in polychrome (visually contrastive) than in monochrome (visually neutral) contexts (Hypothesis 2A). We view this hypothesis as a replication of the effect observed in the literature. It also calls for an extension, as it is needed to make a further comparison between multi-cardinality (visually contrastive) vs. one-cardinality (visually neutral) contexts in terms of cardinality over-specification. The over-specification of small (2 to 4) cardinalities is expected to be higher in multi-cardinality contexts than in one-cardinality contexts (Hypothesis 2B). Secondly, the over-specification of borderline (5–8) cardinalities is expected to be higher in an ordered design than in an unordered design (Hypothesis 2C). The reason is that, when ordered, such cardinalities are presented in small groups that undergo subitization. This effect vanishes when such cardinalities are given in an unordered fashion.

Hypothesis 3 deals with the modifier position and is divided into Hypotheses 3A and 3B. According to Hypothesis 3A, the prenominal position is more typical than the postnominal position for color adjectives in referential communication. According to Hypothesis 3B, the prenominal position is more typical than the postnominal position for numerals. Both hypotheses are verified on Russian data and are motivated by the basic linear word order for Russian numeral phrases and noun phrases with adjectival modifiers (WALS; Greenberg (ed.), 1963; Bivon, 1971; Dryer, 2013a,b).

Primarily relying on Tarenskeen et al. (2015), Hypothesis 4 says that the speaker adheres to either minimal specification or over-specification of colored objects of small (2–4) cardinalities or of uncolored objects of borderline (5–8) cardinalities throughout a whole communication.

## 3 First experiment: small cardinalities and color

### 3.1 Participants

Fifty native Russian speakers were voluntarily recruited for both conditions of the first experiment: twenty-five participants per condition. All participants confirmed to have normal or corrected normal vision and lack of color blindness. The participants gave permission to audio-record their responses.

In the Same Color and Cardinality Condition, the ages of the participants ranged from 19 to 45 years, with the mean age 23.7 years. There were 5 males and 20 females. In the Different Color and Cardinality Condition, the ages of the participants ranged from 18 to 39 years, with the mean age of 25.6 years. There were 24 females and one male.

### 3.2 Methods

#### 3.2.1 Design and materials

The first experiment had a between-subjects design (Same Color and Cardinality condition vs. Different Color and Cardinality condition) and a within-subjects design for each condition (color vs. cardinality). The aim was to compare over-specification rates of color and cardinality in neutral vs. contrastive contexts. Critical and filler items were created on the Overleaf platform for both Same/Different Color and Cardinality conditions. Each slide contained a 2 × 2 grid such that each cell had various geometric figures. The figures differed between all four cells but were identical in each cell. A target cell was highlighted. Any cell could be a target.

In the Same Color and Cardinality condition, all the cells on a slide were of the same color and cardinality. The experimental materials were identical to the ones of the second experiment reported in Zevakhina et al. (2021). The only difference between the experiments lies in the design: the items in the present experiment were given in flash mode, and the items in the second experiment (Zevakhina et al., 2021) were given in non-flash mode. There were four colors (red, blue, yellow, and green) and three cardinalities (two, three, and four). As a result, the condition had 48 critical slides: 4 figures × 4 colors × 3 cardinalities.

In the Different Color and Cardinality condition, each cell on a slide differed from the rest in color and cardinality. There were also four colors (red, blue, yellow, and green) and four cardinalities (two, three, four, and five). It is important to emphasize that we used cardinality five in this condition because of the design. Each cell had to have a different color and cardinality, that is, four colors and four cardinalities. We could not use the cardinality one because it is linguistically expressed with a singular form of a noun in contrast to other cardinalities expressed with plural forms of a noun. For example, one triangle can be expressed by saying *a triangle*, whilst three or five triangles can be expressed by saying *triangles* in the plural form. However, we were aware that cardinality five does not belong to a well-acknowledged subitizing diapason 2–4. We verified whether the results obtained for cardinality five differed from the results obtained for cardinality four, see Section 3.3. Five figures were presented in a dice manner throughout the entire experiment, that is, they were ordered (see Section 4 for the ordered vs. unordered design of the second experiment that tested cardinalities 5–8). This suggested an increase in the number of critical slides: 4 figures × 4 colors × 4 cardinalities = 64. We decided to avoid this because the video presentation might have been too long and tiresome for the participants. The number of critical slides was limited to 48 analogously to the Same Color and Cardinality condition. Sixteen out of 64 critical slides were excluded, which resulted in that each cardinality occurred 48/4 = 12 times for each geometric form, and there were three iterations for each of the four cardinalities (two, three, four, and five).

We expected to receive the following types of responses: a bare plural form of a noun (e.g., ‘triangles’), numeral + noun (e.g., ‘four triangles’), color adjective + noun (e.g., ‘blue triangles’), numeral + color adjective + noun (e.g., ‘four blue triangles’).

**Figure 1:**
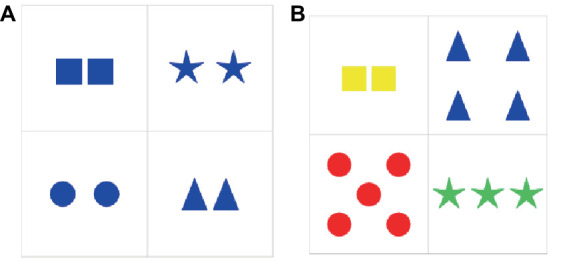
Examples of critical items used in the first experiment: **(A)** Same Color and Cardinality condition (left); **(B)** Different Color and Cardinality condition (right).

Examples of critical items used in both conditions are given in Figure 1.

**Figure 2:**
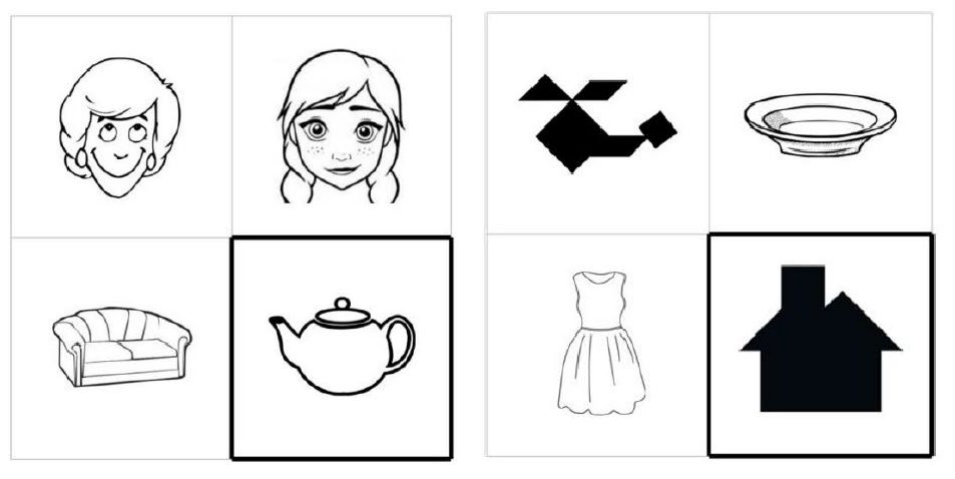
Examples of filler items used in the first experiment. The fillers of the present study are identical to those used in Zevakhina et al. (2021), they are available via the link: https://osf.io/xfrj7/.

In addition to critical items, the experiment had 72 filler items identical in both conditions and similar to the ones used by Tarenskeen et al. (2015). They included human faces, tangrams, and artifacts (crockery, furniture, transport, and clothing). The idea was that the artifacts were the easiest to describe since their identification usually does not take much effort. The tangrams were the most difficult to describe, since they were unusual images and suggested various interpretations. Human faces were in-between and had many features to concentrate on, such as with vs. without beard, with vs. without glasses, hair style, mood of a person, dress, etc. (see Koolen et al., 2013). The fillers were black-and-white, so the participants did not include color in their descriptions. The fillers were identical to those used in the studies reported in Zevakhina et al. (2021). Examples of filler items are shown in Figure 2.

#### 3.2.2 Procedure

Сritical and filler items were counterbalanced and randomly ordered. Every two critical items were separated with at least one filler.

**Figure 3:**
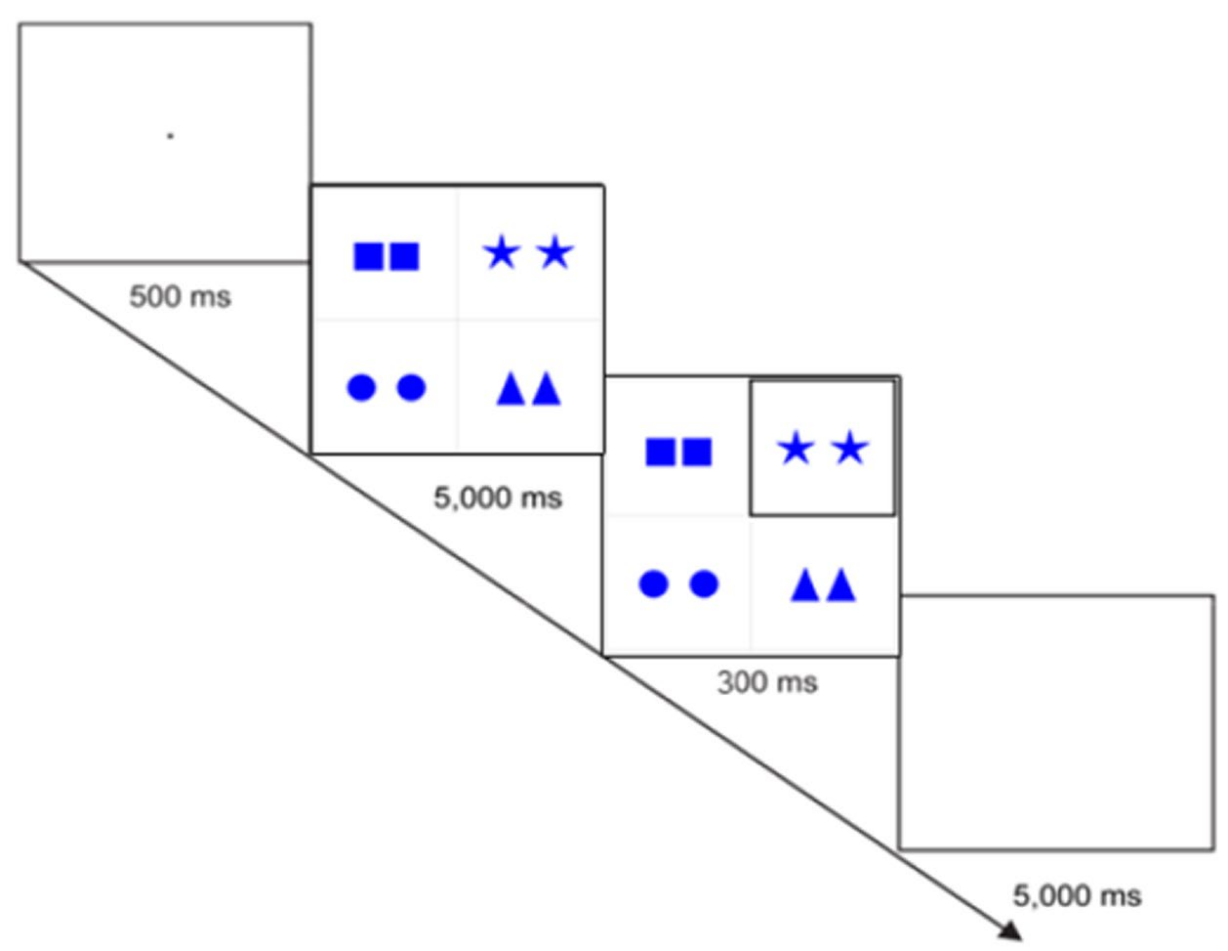
Procedure used in the first experiment (Same Color and Cardinality Condition).

For both conditions of the first experiment, the presentation contained 120 critical and filler slides that were transformed into a video presentation with the help of PowerPoint software. The slides of a video were presented to the participants in flash mode. Each slide had a cycle of some additional slides that appeared in the following order (see Figure 3 for the Same Color and Cardinality Condition). A slide with a dot in the center presented for 500 ms was followed by a slide with geometric figures presented for 5,000 ms. The next slide contained the same figures, with a highlighted target cell, and was presented for 300 ms. After that, a blank slide appeared for 5,000 ms. All in all, each condition of the first experiment contained 480 slides. The experiment took approximately 22 minutes to complete.

The experiment was conducted online via Zoom and was audio and video recorded. Each participant responded to only one condition, was instructed to carefully observe all four cells, to pay attention to which of them would be highlighted, and to describe a target cell to an experimenter who would see the same four cells in a different order. The participants were asked not to refer to cell locations and were informed of the time limit for each slide.

It is important to explain the timing of the slide with a target cell (300 ms). In the third experiment reported in Zevakhina et al. (2021), a target slide was presented for 200 ms that was relatively quick for the participants to subitize a highlighted cell with four objects maximally, with one object subitized for 70 ms, which is an average according to Trick (1992). The first experiment of the present study increased the timing to 300 ms, which covers and even slightly exceeds an average timing (280 ms) for subitizing four objects. Even though one of the conditions included five objects in a cell, it still used the same timing of 300 ms. The motivation for this was to directly compare the results of two experimental conditions of the first experiment. In addition, the five objects were presented in a dice (ordered) manner, so that the objects might be subitized in groups (e.g., two and three objects), which is impossible in numerosity ranges greater than 10.

Let us now motivate the use of the five-second interval at the beginning of the experiment of the present study. It was chosen analogously to the third experiment reported by Zevakhina et al. (2021) where for the first time we tested over-specification of small (2–4) cardinalities with an emphasis on subitizing effects. The procedure was quite similar to the one used in the present study. A slide with a dot (to draw the participants’ attention) was given for 500 ms and followed by a sequence of slides: (i) a slide with all uncolored (up to four) objects presented for 5 sec; (ii) the same slide with a highlighted cell presented for 200 ms; (iii) a blank slide given for 5 sec (to allow the participants to describe a highlighted cell). The aim of presenting the objects for 5 sec at the beginning was to allow the participants to carefully examine the slide. This was necessary to control the over-specification of a small cardinality. Suppose the opposite: there would be no five-second slide with all the objects and a slide with a highlighted cell would be straightforwardly presented. In that case, the participants could subitize a cardinality, but we could not verify the idea that their responses were over-specified, because over-specification is determined by a surrounding context. So, the participants were presented with all the objects for 5 sec. Could this interval be sufficient to count all the objects? Yes, that could be sufficient, but we do not see the reason for why this should be necessary to count all the objects in all the cells. This seems to be redundant for the experiment’s purpose and, therefore, implausible.

### 3.3 Results

#### 3.3.1 Over-specification of color and small cardinalities

**Figure 4:**
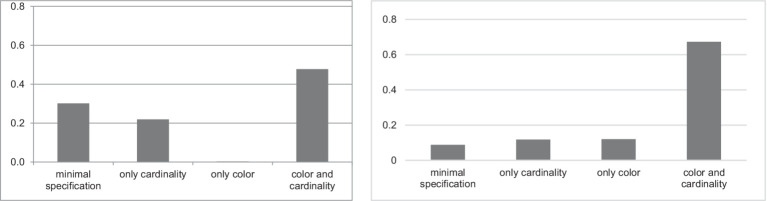
Distribution of the responses in the Same Color and Cardinality condition (left) vs. in the Different Color and Cardinality condition (right) in the first experiment.

We collected 1,192 responses for the critical items in the Same Color and Cardinality condition. Eight of them (0.67%) were excluded for the following reasons: two errors in color identification, one error in figure identification, and five technical errors (some participants either did not notice which cell was highlighted or were distracted). Among the 1,192 responses, 359 (30.1%) were minimally specified, that is, they did not include color or cardinality identification. Most of the responses (569, or 47.7%) were over-specified both in color and in cardinality, while 261 responses (21.9%) were over-specified in cardinality, and three responses (0.25%) were over-specified in color. The distribution of the responses is presented in Figure 4.

We collected 1,188 responses for the critical items in the Different Color and Cardinality condition. Twelve responses (1.01%) were excluded for the following reasons: an error in metaphorical naming of figures, two errors in color identification, four errors in cardinality identification, one error in figure identification, and four technical errors (some participants did not notice which cell was highlighted, were distracted, or there was poor Internet connection). Among the 1,188 responses, 105 (8.8%) were minimally specified, i.e., they did not include color or cardinality over-specification. One hundred forty responses (11.8%) redundantly specified only cardinality, while 143 responses (12%) redundantly specified only color and 800 (67.4%) redundantly specified both color and cardinality. The distribution of the responses is given in Figure 4.

Using the R Core Team (2020), we performed the McNemar test that demonstrated a significant difference between cardinalities and color in terms of over-specification vs. minimal specification in the Same Color and Cardinality condition (*χ^2^* = 250.19, *df* = 1, *p* < 0.0001). This confirms Hypothesis 1. However, the McNemar test did not reveal any differences between cardinalities and color in terms of over-specification vs. minimal specification in the Different Color and Cardinality condition (*χ^2^* = 0.014134, *df* = 1, *p* = 0.9). Thus, both cardinality and color were over-specified at similar rates in polychrome contexts. This does not confirm Hypothesis 1.

Before moving on, let us consider the role of presence/absence of an interlocutor. The responses from one participant in the Same Color and Cardinality condition and from six participants in the Different Color and Cardinality condition were received without an experimenter when the participants could not arrange a Zoom call due to their personal circumstances. These responses were not excluded from the statistical analyses. It is interesting to see whether these responses differed from the rest. All the responses from one participant in the Same Color and Cardinality condition (except for the first three responses – therefore, 45 responses, that is, 4% of the 1,192 responses) and all the responses from two participants in the Different Color and Cardinality condition (288 responses, that is, 24% of the 1,188 responses) were both color and cardinality over-specified. Recall that most of the responses (569, that is, 47.7% of the 1,192 responses) were over-specified both in color and cardinality in the Same Color and Cardinality condition; and most of the responses (800, that is, 67.4% of the 1,188 responses) redundantly specified both color and cardinality in the Different Color and Cardinality condition. The latter data point out that color and cardinality over-specification responses received without an experimenter in the Different Color and Cardinality condition constituted 36% part of all the responses received in that condition. Although the question of the potential influence of the presence/absence of an interlocutor on the over-specification of properties was not addressed in the present study, we can say that we are unable to draw any solid implications from these results. This question deserves further investigation.

#### 3.3.2 Additional over-specifications

We expected that there would be three ways of giving redundant descriptions: only cardinality (e.g., two circles), only color (e.g., red stars), cardinality and color (e.g., three green triangles). However, some participants provided additional over-specifications of figure (e.g., a red five-pointed star or three triangles…isosceles) and location (e.g., four red triangles forming a square, three red stars located in a horizontal row, two blue circles near each other) in both conditions. All participants who generated additional over-specifications were asked to repeat an instruction given before the experiment to make sure that they were correctly performing the task. All participants repeated the task, demonstrating that they had understood it. They commented on their behavior, stating that it was easier for them to describe everything they could. However, they had the feeling that they were obliged to do so. These comments indicate that the maximally specified descriptions were speaker-oriented rather than listener-oriented. There were 119 responses (approximately 10%) with maximal over-specifications, four responses (0.3%) with cardinality, and additional over-specifications in the Same Color and Cardinality condition. There were 235 responses (approximately 20%) with maximal over-specifications, five responses (0.4%) with cardinality and additional over-specifications, and one response (0.08%) with color and additional over-specifications in the Different Color and Cardinality condition. Moreover, all the responses from four participants in the Different Color and Cardinality condition (192, that is, 16% of the 1,188 responses), which were received without an experimenter, included additional redundant specifications of figures and/or location. Recalling that there were 235 responses (approximately 20% of the 1,188 responses) with maximal over-specifications, this is an interesting result and deserves further investigation of potential influence of the presence/absence of an interlocutor on the over-specification of properties in referential communication.

**Figure 5:**
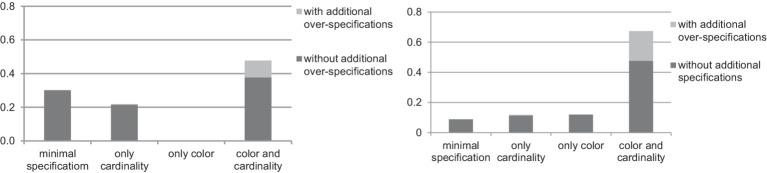
Distribution of the responses in the Same Color and Cardinality condition (left) vs. in the Different Color and Cardinality condition (right) with additional over-specifications in the first experiment.

The distribution of the responses with/without additional specifications in both conditions is presented in Figure 5.

#### 3.3.3 Visual contexts for color and small cardinalities

**Figure 6:**
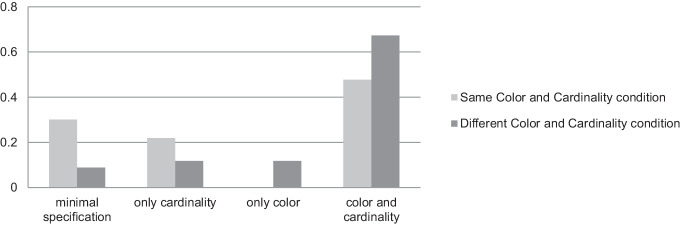
Results for the Same vs. Different Color and Cardinality conditions in the first experiment.

Regarding the neutral vs. contrastive visual context, we performed the Wilcoxon rank sum test that showed that color over-specification was much higher in the polychrome context than in the monochrome one (*W* = 485,788, *p* < 0.0001), see Figure 6. This confirms Hypothesis 2A.

As seen in Figure 6, cardinality over-specification was higher in the Different Color and Cardinality condition than in the Same Color and Cardinality condition (*W* = 640,828, *p* < 0.0001). This confirms Hypothesis 2B. Why is it so?

One plausible reason is that cardinality five was used in the Different Color and Cardinality condition, while the Same Color and Cardinality condition lacked it and included only cardinalities 2–4. Did cardinality five differ from the other cardinalities? We compared over-specification and minimal specification rates for cardinalities four and five in the Different Color and Cardinality condition using binary logistic regression with items and subjects as random factors. It turned out that the difference between them was not significant (*β* = 1.1990, *SE* = 0.6552, *z* = 1.830, *p* = 0.0673). This result is surprising because cardinality five seems to be more difficult to perceive than cardinalities 2–4. However, the result indicates that the frame timing of 300 ms (see Figure 3) was sufficient even for cardinality five. Recall that five objects were presented in dice order throughout the whole experiment.

Another plausible reason is that the Different Color and Cardinality condition provided contrastive contexts that boosted color and cardinality over-specification rates and diminished probabilities of producing other descriptions. We take this explanation as more reasonable than the former one.

#### 3.3.4 Position of numerals and color adjectives

**Figure 7:**
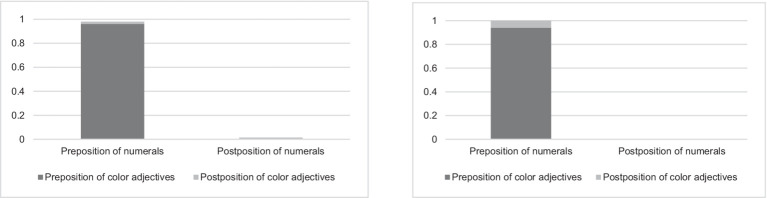
Position of color adjectives and numerals in the Same Color and Cardinality condition (left) vs. in the Different Color and Cardinality condition (right) in the first experiment.

The results revealed the following patterns for the position of numerals and color adjectives. Consider the Same Color and Cardinality condition. All the responses with only color or only cardinality over-specification included prenominal numerals or color adjectives. In the responses with both color and cardinality over-specification, prenominal modifiers predominated (see Figure 7). McNemar test did not show significant differences between color adjectives and numerals in terms of position (*χ^2^* = 3.5, *df* = 1, *p* = 0.06). This suggests that both color adjectives and numerals take prenominal positions and confirms Hypotheses 3A and 3B.

It is interesting to highlight the differences in the rest of the patterns. It is more likely that a color adjective would take a postposition compared to a numeral. When a numeral takes a postnominal position, it is more likely to be accompanied by the noun *štuka* ‘thing’ which serves as a kind of classifier, *cf.* (12) and (13).

12. *Dva kvadrata žoltykh*.

two square.GEN.SG yellow.GEN.PL.

‘Two yellow squares.’

13. *Zeljonye zvjozdy četyre^??^(štuki)*.

Green.NOM.PL square.NOM.PL four thing.NOM.PL.

‘Four green stars.’

In the Different Color and Cardinality condition, all the responses with only cardinality over-specification included prenominal numerals, while 137 responses (approximately 93%) with only color over-specification included prenominal color adjectives and the rest (approximately 7%) included postnominal color adjectives. Prenominal modifiers prevailed in the responses with both color and cardinality over-specification (see Figure 7). McNemar test indicated a significant difference between color adjectives and numerals in terms of position (*χ^2^* = 44.022, *df* = 1, *p* < 0.0001). This suggests that even though the prenominal position for color adjectives is predominant, the postnominal position is also possible, while numerals can only take the prenominal position. This confirms Hypothesis 3B and partially confirms Hypothesis 3A.

#### 3.3.5 Consistency

**Figure 8:**
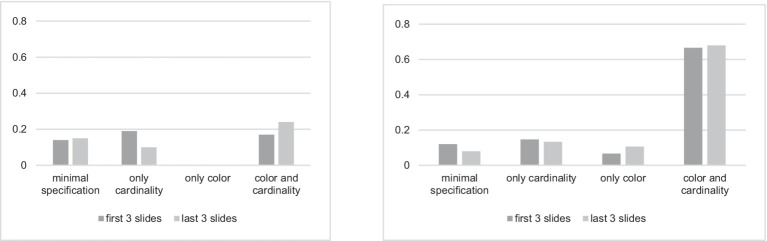
Distribution of the responses in the Same Color and Cardinality condition (left) vs. in the Different Color and Cardinality condition (right) for the first 3 and the last 3 slides in the first experiment.

Consistency and switching between consistency strategies are also worth discussing. The distribution of the responses in the first three slides and in the last three slides in both conditions is shown in Figure 8.

The cardinality over-specification prevailed in the beginning of the experiment in the Same Color and Cardinality condition. The color and cardinality over-specification increases to the end of the experiment. This suggests that the participants were initially inclined to recognize cardinality much more often than color, but then they could also include color in their descriptions. The minimal specification strategy turned out to be the most stable and it quantitatively increased toward the end of the experiment. This means that if the participants initially chose it, they less rarely changed it to other strategies. The color over-specification occurred neither in the beginning nor toward the end of the experiment and had quite few occurrences in the middle.

In the Different Color and Cardinality condition, the switching between the strategies was less considerable than in the other condition. Both color and cardinality over-specification prevailed and did not seem to change during the experiment.

An average rate of consistency in the Same Color and Cardinality condition was 91.5%. Eleven participants (44%) demonstrated consistency in 100% of their responses, while 20 participants (80%) showed consistency in more than 80% of their responses, and the lowest value was 60.4%. An average rate of consistency in the Different Color and Cardinality condition was 88.7%. Ten participants (40%) showed consistency in 100% of their responses, while 20 participants (80%) demonstrated consistency in more than 80% of their responses, and the lowest consistency value was 42.2%. Therefore, in both conditions, 80% of the participants demonstrated consistency in over-specification in more than 80% of the responses. This confirms Hypothesis 4.

### 3.4 Discussion

As the results of the first experiment showed, Hypothesis 1 was supported in the Same Color and Cardinality condition but not in the Different Color and Cardinality condition. The results for the Same Color and Cardinality condition accord with the results of the second experiment reported in Zevakhina et al. (2021) since the experimental materials were identical. The only difference between the two experiments lies in the design: the items were presented in flash mode in the present study and in non-flash mode in the previous one. This provides additional evidence for that small (2–4) cardinalities are more salient (due to subitizing) than color in visually neutral contexts.

The contrast between the Same vs. Different Color and Cardinality conditions corroborated Hypotheses 2A and 2B. Not only this result provided further evidence for the distinction between monochrome vs. polychrome contexts observed in the literature (Belke and Meyer, 2002; Koolen et al., 2013; Rubio-Fernandez, 2016; Long et al., 2021; Rubio-Fernandez et al., 2021), but also, more importantly, it discovered a distinction between one-cardinality vs. multi-cardinality contexts. Cardinality over-specification rates are higher in multi-cardinality contexts than in one-cardinality contexts. This provides additional evidence for that small (2–4) cardinalities are more salient (due to subitizing) than color in visually contrastive contexts.

Hypotheses 3A and 3B were supported in the Same Color and Cardinality condition, but only Hypothesis 3B was supported in the Different Color and Cardinality condition, and Hypothesis 3A was partially confirmed. In both conditions, both types of modifiers took not only the prenominal position, but also (quite rarely) the postnominal position. The articulation of both modifiers in one message is of special interest. In the Same Color and Cardinality condition, postnominal positions of any modifier did not give a significant difference. In the Different Color and Cardinality condition, color adjectives were used in the postnominal position more often than numerals, as though color was considered less salient than cardinality and as supplementary information. This is a puzzling result, since the condition is contrastive in terms of color, and contrastive polychrome contexts are usually salient and increase over-specification rates of color. This reinforces the contrast between color and small (2–4) cardinalities in terms of salience. Small cardinalities are more salient (due to subitizing), and consequently, numerals are immediately reported. On the contrary, color is less salient compared to small (2–4) cardinalities and therefore color adjectives can be disfluent if reported. It is worth noting again that this disfluency is rare.

Hypothesis 4 was supported. The participants were consistent in either over-describing or minimally describing objects throughout the whole communication. It is worth noting that the neutral contexts revealed slightly higher percentages of consistency and less switching between strategies than the contrastive contexts.

All these findings imply that cardinality as a property is more salient than color in visually neutral, or non-contrastive, contexts. However, color salience increases in contrastive contexts, with cardinality salience not changing that much. However, in such contexts, cardinality seems to be slightly more salient than color due to the occasional disfluency of color adjectives.

## 4 Second experiment: borderline cardinalities

### 4.1 Participants

Fifty native Russian speakers were voluntarily recruited in both conditions of the second experiment. Twenty-five participants (16 females and 9 males) responded to the Ordered condition. Their ages ranged from 18 to 29, with the mean age of 21.3 years. Twenty-five participants (17 females and 8 males) responded to the Unordered condition. Their ages ranged from 18 to 26, with the mean age of 21.6 years. All the participants gave permission to audio-record their responses and had normal or corrected to normal vision.

### 4.2 Methods

#### 4.2.1 Design and materials

**Figure 9:**
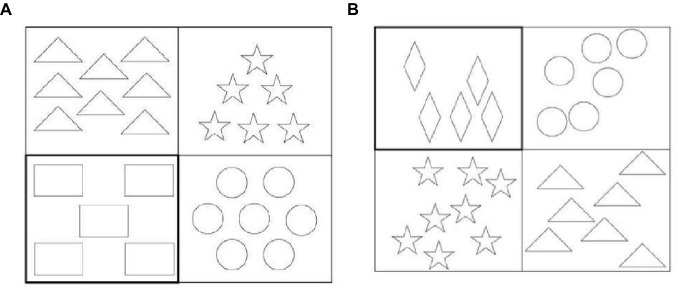
Examples of critical items used in the second experiment: **(A)** Ordered Cardinality condition (left); **(B)** Unordered Cardinality condition (right).

The second experiment tested cardinalities ranging from 5 to 8 and had a between-subjects design (Ordered vs. Unordered conditions). For both conditions, critical and filler items were created using Canva software. The design of the second experiment resembled the design of the first one. The critical items comprised the following geometric figures: squares, rectangles, circles, ovals, triangles, stars, and diamonds. There were 32 critical slides. Each slide included a 2 × 2 grid. Each cell had 5–8 identical figures. Figures varied between cells. A target cell was highlighted. Any cell could be a target. Figure 9 illustrates examples of critical items used in the second experiment.

Forty-eight fillers were a subset of those used in the first experiment: human faces, tangrams, and artifacts. The critical and filler items were counterbalanced and randomly ordered. However, every two critical items were separated with at least one filler.

#### 4.2.2 Procedure

**Figure 10:**
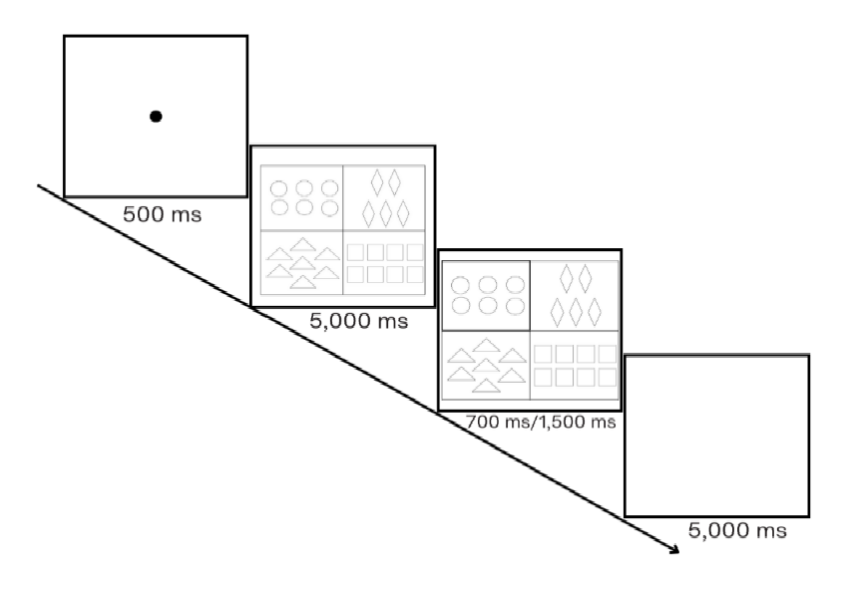
Procedure used in the second experiment (Ordered Cardinality condition).

For both conditions, the presentation contained 80 critical and filler slides that were transformed into a video presentation. The presentation was made using the Overleaf platform, while its video version was created with the help of the WPS office. The slides of a video were presented to the participants in flash mode. Each slide had a cycle of some additional slides that appeared in the following order (see Figure 10 for the Ordered Cardinality condition). A slide with a dot in the center presented for 500 ms was followed by a slide with geometric figures presented for 5,000 ms. The next slide contained the same figures, with a highlighted target cell, and was presented for 700 ms in the Ordered Cardinality condition, which almost reaches the maximum 800 ms of time phase to estimate 8 objects. In the Unordered Cardinality condition, such a slide was presented for 1,500 ms, which is slightly more than 2 × 700 ms for the ordered condition. We believe that, on the one hand, objects presented in a random way are more difficult to perceive than objects presented in some order and, therefore, require more time to be estimated. On the other hand, we did not want to allocate too much time to estimate a given set of objects. After that, a blank slide appeared for 5,000 ms. In total, each condition of the first experiment contained 320 slides. The experiment took approximately 20 minutes to complete.

The experiment was conducted online via Zoom and was audio and video recorded. Each participant responded to only one condition and was instructed to describe a target cell in a slide to an experimenter who had the same set of slides, but the cells in each slide were presented in a different order. The participants were asked not to refer to cell locations. The participants also knew about a time limit for each slide.

### 4.3 Results

#### 4.3.1 Over-specification of (un)ordered borderline cardinalities

We expected 1,600 responses for the critical items. However, 29 responses (1.8%) were excluded due to technical errors during the experiment. Next, 21 other responses (1.3%) were also excluded because of the following reasons: errors in cardinality identification, imprecise responses (e.g., triangles seven or eight), responses with detailed location of ordered figures (e.g., squares four together and one separately, two triangles at the top and four triangles at the bottom, two rows of stars, squares three things in two rows). From the last category, we can conclude that the figures were perceived in parts with 2–4 objects to be subitized.

**Figure 11:**
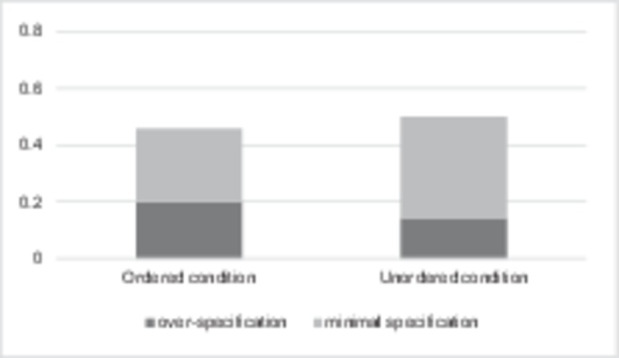
Distribution of the responses in the Ordered Cardinality vs. Unordered Cardinality conditions in the second experiment.

Overall, there were 1,550 responses for the critical items in both conditions: 769 responses (49.6%) in the Ordered Cardinality condition and 781 responses (50.4%) in the Unordered Cardinality condition. Among them, 399 responses (51.9%) were minimally specified in the Ordered Cardinality condition, and 566 responses (72.5%) were minimally specified in the Unordered Cardinality condition. The distribution of the responses is presented in Figure 11.

Using R Core Team (2020), we performed the Wilcoxon rank sum test that demonstrated a significant difference between Ordered Cardinality vs. Unordered Cardinality conditions in terms of minimal specification vs. over-specification responses (*W* = 226,606, *p* < 0.0001). This suggests that the Ordered Cardinality condition facilitates over-specification of borderline (5–8) cardinalities much more than the Unordered Cardinality condition. This confirms Hypothesis 2C.

#### 4.3.2 Additional over-specifications

Two ways of delivering redundant descriptions were expected to be given: only cardinality (e.g., two circles) or no cardinality (e.g., circles, many circles).

The over-specification of additional properties occurred much rarer in the second experiment than in the first one. In the Ordered condition, there were four responses (0.5% of the 769 responses collected for this condition) provided by two participants that included white color (all the figures were uncolored): e.g., seven white triangles. One of those participants specified the vertical location of the figures two times (0.3%): for example, seven vertical diamonds. In the Unordered condition, there were seven responses (0.9% of the 781 responses collected for this condition) provided by one participant that included white color (all figures were uncolored). One participant once specified a shape (five elongated ovals; 0.13%) and another participant specified a size (five not high triangles; 0.13%).

#### 4.3.3 Position of numerals

**Figure 12:**
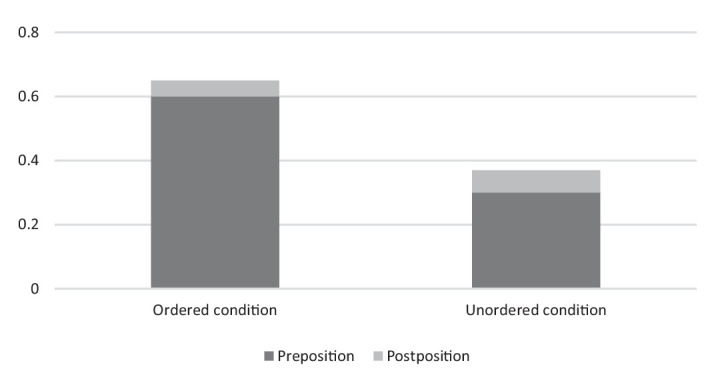
Position of numerals in the Ordered Cardinality vs. Unordered Cardinality conditions in the second experiment.

Regarding the position of the numerals, McNemar test demonstrated a significant difference between the Ordered Cardinality and Unordered Cardinality conditions in terms of preposition vs. postposition of the numerals (*χ^2^* = 100.24, *df* = 1, *p* < 0.0001). It means that the prenominal position of the numerals is dominated for both ordered and unordered figures; however, the postnominal position of the numerals is more likely to occur in descriptions of unordered figures. This partially confirms Hypothesis 3B. See also Figure 12.

The fact that the prenominal numerals comprised 88% indicated that the participants easily construed their responses and considered the over-specified numerals to be important to communicate. Additionally, it is important to note that some responses included approximative constructions and modal adverbs such that both groups involve postnominal numerals, *cf.* (14)–(16).

14. *Treugol’niki štuk sem’.*

triangle.NOM.PL thing.GEN.PL seven.

‘Triangles, seven things.’

15. *Mnogo prjamougol’nikov vrode šest’.*

many rectangle.GEN.PL probably six.

‘Many rectangles, probably six.’

16. *Rombiki šest’ naverno.*

diamond.NOM.PL six probably.

‘Diamonds six probably.’

Such modal adverbs occurred predominantly in the responses of seven participants (14%): 14 responses (1.8%) in the Unordered condition and six responses (0.78%) in the Ordered condition. Therefore, even though the participants were not sure that the cardinality was correct, they intended to specify a cardinality of objects that appeared on the screen. The fact that the postnominal numerals comprised 12% and occurred only in approximative and modal constructions indicated that the participants were not sure in their responses and considered the over-specified numerals as supplementary information.

#### 4.3.4 Consistency

Consistency was also observed in the second experiment, but it has a smaller effect than in the first experiment. In the Unordered Cardinality condition, 32% of the participants over-specified borderline (5–8) cardinalities in most of their responses (65–100%), and 60% of the participants minimally described borderline (5–8) cardinalities in most of their responses (91–100%). In the Ordered Cardinality condition, 44% of the participants used over-descriptions in most of their responses (64–100%), while 52% of the participants minimally specified borderline (5–8) cardinalities in most of their responses (63–100%). These results indicate that some of the participants adhered to one strategy during a whole experiment: either to over-specify or to minimally specify cardinalities. However, the percentages of the participants who followed one strategy were not greater than 60%. On the contrary, the percentages of such participants in the first experiment were greater than 80%. All said above suggests that the results of the second experiment only partially confirm Hypothesis 4. It is interesting to point out that the minimal specification strategy prevailed in the second experiment, while the over-specification strategy dominated in the first experiment.

**Figure 13:**
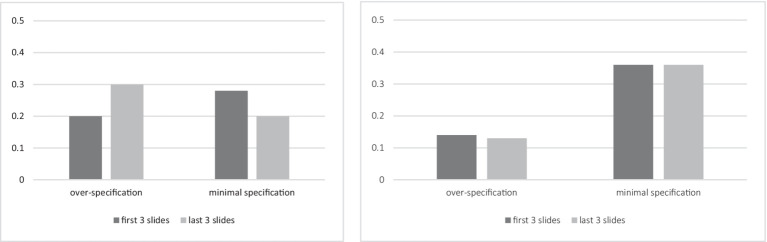
Distribution of the responses in the Ordered condition (left) vs. Unordered condition (right) for the first 3 and the last 3 slides in the second experiment.

The distribution of the responses on the first 3 and the last 3 slides of both conditions presented in Figure 13 shows the following. In the Ordered condition, the cardinalities were more likely to be minimally specified in the beginning of the experiment, while they were more likely to be over-specified in the end of the experiment. In the Unordered condition, the cardinalities were more likely to be minimally specified at the beginning and end of the experiment.

### 4.4 Discussion

The results of the second experiment confirmed Hypothesis 2C, demonstrating that the perception of borderline (5–8) cardinalities depends on the order of objects. If objects are displayed in an ordered way, they are likely to be over-specified. If objects are randomly displayed, they are more likely to be minimally described.

Partial confirmation of Hypothesis 3B suggests the following. When over-specified, the numerals of ordered and unordered borderline (5–8) cardinalities predominantly took prenominal positions, and postnominal positions rarely occurred. However, numerals denoting unordered (5–8) borderline cardinalities were more frequently used postnominally than numerals denoting ordered ones.

The partial confirmation of Hypothesis 4 provides evidence for the following. Firstly, consistency in over-specification was observed to a greater extent for ordered objects than for unordered objects. Secondly, consistency in over-specification was more notable for small (2–4) cardinalities in the first experiment than for borderline (5–8) cardinalities in the second experiment.

All said above leads to the following implications. Borderline (5–8) cardinalities are less salient than small (2–4) cardinalities because, generally, they involve estimation rather than subitization, thus yielding less over-specified descriptions. However, ordered cardinalities might involve another strategy. When ordered, borderline (5–8) cardinalities can be perceived in small groups (2–4 items per group), and such groups can be subitized. This explains why over-specified messages were more frequent for ordered than unordered cardinalities. In contrast, when unordered, borderline (5–8) cardinalities are difficult to group and, therefore, they are less likely to be subitized. They seem to be estimated, resulting either in minimal specification of objects (without delivering numerals) or in postnominal positions of numerals. People could start their messages with figure names and add numerals when they estimated cardinalities in their minds.

## 5 General discussion

Over-specification of properties in referential communication is still a relatively new area of multidisciplinary research. Despite its novelty, some topics have been thoroughly investigated. In what follows, we name only some of them that are most relevant to the present study. Firstly, color has been acknowledged to be an absolute and very salient property, thus yielding much higher over-specification rates than any other property: size, shape, pattern, etc. (cf. Belke and Meyer, 2002; Engelhardt et al., 2006; Arts et al., 2011; Brown-Schmidt and Konopka, 2011; Koolen et al., 2011; Gatt et al., 2013; Van Gompel et al., 2014; Tarenskeen et al., 2015; Rubio-Fernandez, 2016, 2019, 2021; Long et al., 2021; Rubio-Fernandez et al., 2021; among others). Moreover, its over-specification has been claimed to be dependent on discriminability of surrounding environment: polychrome contexts, or higher color discriminability, give rise to higher over-specification rates than monochrome contexts, or lower color discriminability (*cf.* Belke and Meyer, 2002; Koolen et al., 2013; Rubio-Fernandez, 2016; Long et al., 2021; Rubio-Fernandez et al., 2021; among others). Secondly, strategies in referring to objects have been demonstrated to be consistent and to involve either over-specification or minimal specification (Tarenskeen et al., 2015; among others). Thirdly, language syntax has been argued to determine the position of modifiers that linguistically encode properties: (color) modifiers are more frequent in languages with prenominal position than in languages with postnominal position (Rubio-Fernandez, 2016; Rubio-Fernandez, 2019; Rubio-Fernandez et al., 2021). Fourthly, small (2–4) cardinalities have been demonstrated to be over-specified even more than color due to its greater salience caused by the subitizing effect (Zevakhina et al., 2021).

The present study primarily focused on cardinalities (and numerals) in the aspects given above: salience, visual context, consistency, modifier position, and subitizing. This is, on the one hand, an extension of the acknowledged effects studied now on a new material and, on the other hand, the discovery of some new effects. All these aspects are discussed in the following.

The present study replicated the findings by Zevakhina et al. (2021): small (2–4) cardinalities are more salient (due to subitizing) than color and therefore are over-specified to a greater extent than color. Moreover, as the present study pointed out, they demonstrate less dependency upon visual context than color. Over-specification rates of small (2–4) cardinalities varied to a lesser extent than over-specification rates of color in neutral vs. contrastive contexts. A plausible reason is that the subitizing effect is preserved in both types (one-cardinality and multi-cardinality) of contexts, and, therefore, the salience of small (2–4) cardinalities is high. However, what is interesting is the question of which factor plays an important role in contrastive contexts that increase over-specification rates of small (2–4) cardinalities: multi-cardinality, higher color discriminability, or both. This question needs further research.

The present study provided evidence that borderline (5–8) cardinalities generally involve estimation rather than subitizing. This results in lower over-specification rates. However, the study also discovered a new factor that determines the over-specification rates of borderline (5–8) cardinalities. It is the order. When borderline (5–8) cardinalities are ordered, they are displayed in small groups that are likely to be subitized, and, therefore, their over-specification rates increase. When they are unordered, they are estimated rather than subitized, and, consequently, their over-specification rates decrease. It seems that the order factor concerns not only the borderline (5–8) cardinalities, but also some larger cardinalities. The question to investigate is what the role of order in larger cardinalities is.

The present study stated that the well-known consistency factor is preserved in both types (small and borderline) of cardinalities. Interestingly, small (2–4) cardinalities are consistent in their relatively high over-specification rates, while borderline (5–8) cardinalities demonstrate a less stable consistency in their over-specification. The latter fact is explained by the diminishing effect of subitizing in borderline (5–8) cardinalities. This effect is even less tangible when borderline (5–8) cardinalities are unordered.

The present study highlighted several issues relevant to the discussion of the modifier position. Firstly, the modifier position can be subject to variation. Even though numerals and color adjectives predominantly take prenominal positions in the language under consideration, both types of modifiers can also take postnominal positions. This suggests that cardinalities and color are predominantly considered important pieces of information to communicate because of their salience. However, manipulations with visual contexts can affect their salience (color is more context sensitive than small cardinalities), resulting in the encoding of them as supplementary information. Secondly, numeral modifiers denoting borderline cardinalities are used in approximative and modal constructions that take postnominal positions. Thirdly, the articulation of numeral modifiers denoting small cardinalities and color adjectives demonstrates that what is more salient precedes what is less salient. This adds to the discussion of modifier position that was primarily focused on cross-linguistic studies (Rubio-Fernandez, 2016; Rubio-Fernandez, 2019; Rubio-Fernandez et al., 2021; among others). Fourthly, the present study observed additional specifications of properties of objects in referential communication. In this respect, the question of whether presence/absence of an interlocutor is a key factor for (additional) over-specification deserves further investigation. To summarize the linguistic contributions of the present study, it is important to note that it did not specifically address linguistic issues of the language under investigation. Rather, the conclusions obtained in the present study seem to be applicable to languages with the following basic word orders: numeral + noun phrase, color adjective + noun phrase.

## 6 Conclusion

The present study made several contributions to our deeper understanding of over-specification of small cardinalities, borderline cardinalities, and color in referential communication. It argued that small (2–4) cardinalities are less dependent on visual context than color, since they are more salient than color due to subitizing in both types (neutral and contrastive) of contexts, thus yielding higher over-specification rates and higher percentages of prenominal positions. The study shed light on the borderline (5–8) cardinalities that are estimated rather than subitized and, therefore, are less salient. They revealed less over-specification rates than small (2–4) cardinalities, and more postnominal uses when over-specified. The order factor plays a key role in their salience, since it suggests subitizing of their small parts. The ordered mode of presentation of borderline (5–8) cardinalities leads to higher over-specification rates and to higher percentages of prenominal positions than the unordered one. The study provided further evidence for the consistency of color, small cardinalities, and borderline cardinalities in referential communication.

## Data availability statement

The datasets presented in this study can be found in online repositories. The repository can be found here: https://osf.io/73jqg/.

## Ethics statement

The studies involving humans were approved by the Institutional Review Board of the National Research University Higher School of Economics. The studies were conducted in accordance with the local legislation and institutional requirements. The participants provided their written informed consent to participate in this study. Written informed consent was obtained from the individual(s) for the publication of any potentially identifiable images or data included in this article.

## Author contributions

NZ: Conceptualization, Formal analysis, Funding acquisition, Investigation, Methodology, Project administration, Supervision, Writing – original draft, Writing – review & editing. KD: Data curation, Investigation, Methodology, Writing – original draft. DS: Data curation, Investigation, Methodology, Writing – original draft. DP: Writing – review & editing.
