# Corrigendum: Over-specification of small, borderline cardinalities and color in referential communication: the role of visual context, modifier position, and consistency

**DOI:** 10.3389/fpsyg.2024.1500005

**Published:** 2024-10-03

**Authors:** Natalia A. Zevakhina, Kseniya N. Dongarova, Daria Shubina, Daria P. Popova

**Affiliations:** HSE University, Moscow, Russia

**Keywords:** over-specification, referential communication, cardinalities, color, modifier position, consistency

In the published article, there was an error. The words “Same” and “Different” should be interchanged, as stated below.

A correction has been made to **3.3.3 Visual contexts for color and small cardinalities**, second paragraph. This sentence previously stated:

As seen in Figure 6, cardinality over-specification was higher in the Same Color and Cardinality condition than in the Different Color and Cardinality condition (W = 640,828, *p* < 0.0001).

The corrected sentence appears below:

As seen in Figure 6, cardinality over-specification was higher in the Different Color and Cardinality condition than in the Same Color and Cardinality condition (W = 640,828, *p* < 0.0001).

The authors apologize for this error and state that this does not change the scientific conclusions of the article in any way. The original article has been updated.

